# Construction and Segmental Reconstitution of Full-Length Infectious Clones of Milk Vetch Dwarf Virus

**DOI:** 10.3390/v17091213

**Published:** 2025-09-05

**Authors:** Aamir Lal, Muhammad Amir Qureshi, Man-Cheol Son, Sukchan Lee, Eui-Joon Kil

**Affiliations:** 1 Department of Integrative Biotechnology, Sungkyunkwan University, Suwon 16419, Republic of Korea; aamirchaudhary43@gmail.com (A.L.); amirq303@gmail.com (M.A.Q.); 2 Department of Plant Medicals, Gyeongkuk National University, Andong 36729, Republic of Korea; sreedom123@naver.com; 3 Agricultural Research Institute, Gyeongkuk National University, Andong 36729, Republic of Korea

**Keywords:** milk vetch dwarf virus, infectious clones, nanovirus, agroinoculation, transient transfection, viral replication

## Abstract

The construction of infectious clones (ICs) is essential for studying viral replication, pathogenesis, and host interactions. Milk vetch dwarf virus (MDV), a nanovirus with a multipartite, single-stranded DNA genome, presents unique challenges for IC development due to its segmented genome organization. To enable functional analysis of its genome, we constructed full-length tandem-dimer-based ICs for all eight MDV genomic segments. Each segment was cloned into a binary vector and co-delivered into *Nicotiana benthamiana*, *Nicotiana tabacum*, *Vicia faba*, and *Vigna unguiculata* plants via *Agrobacterium*-mediated inoculation. Systemic infection was successfully reconstituted in all host plants, with PCR-based detection confirming the presence of all viral segments in the infected leaves of nearly all tested plants. Segmental accumulation in infected plants was quantified using qPCR, revealing non-equimolar distribution across hosts. This study establishes the first complete IC system for MDV, enabling reproducible infection, replication analysis, and quantitative segment profiling. It provides a foundational tool for future molecular investigations into MDV replication, host interactions, and viral movement, advancing our understanding of nanovirus biology and transmission dynamics.

## 1. Introduction

Nanoviruses are non-enveloped, multipartite viruses with circular single-stranded DNA (ssDNA) genomes. They belong to the family *Nanoviridae* and pose significant threats to leguminous and solanaceous crops in various regions of the world [1,2,3,4]. Their complex genome structure, consisting of multiple individually encapsidated DNA segments, presents unique challenges in understanding their lifestyle, i.e., replication, movement, and epidemiology [5]. Their genomes are composed of eight individually encapsidated circular DNA segments, each encoding a single open reading frame (ORF) responsible for a distinct function in the viral life cycle. Unlike geminiviruses, which have monopartite or bipartite genomes with multiple ORFs per segment, nanoviruses follow a strict one-segment-one-gene organization [1,6,7,8]. Among the segments, DNA-R encodes the master replication initiator protein, essential for the rolling-circle replication of all genome components [9,10]. DNA-S encodes the capsid protein (CP), which plays a critical role in genome encapsidation and aphid-mediated transmission [11,12]. DNA-C encodes Clink, a cell cycle link protein that interacts with host retinoblastoma-related proteins, modulating host cell cycling to favor viral replication [13]. DNA-M encodes the movement protein (MP), facilitating the intercellular transport of viral DNA through plasmodesmata, while DNA-N encodes the nuclear shuttle protein (NSP), involved in the nuclear export of viral genomes [4,14]. DNA U2 has been recently reported to act as an RNA-silencing suppressor [15]. The remaining segments, DNA-U1 and DNA-U4, encode proteins with currently unknown functions but are believed to contribute to viral infectivity [1]. The development of Infectious clones (ICs) began with bacteriophages [16] and subsequently adapted for plant viruses to enable precise molecular studies. These enable the study of viral replication, host range, movement, and pathogenesis. The construction of an IC allows researchers to manipulate viral genomes, analyze specific gene functions, and explore the molecular basis of host–virus interactions [17,18,19]. Particularly for insect-transmitted viruses, which cannot be mechanically inoculated, ICs not only facilitate experimental infection without relying on natural vectors but also reduce the risks of genetic drift and mixed infections associated with repeated virus passage in host plants [20,21,22]. Moreover, ICs allow targeted investigations into segment-specific roles, gene expression, and interactions with the host’s silencing machinery [23]. Developing the IC-systems has previously been reported for several ssDNA plant viruses [24] including multipartite viruses like nanoviruses. However, their multipartite genome organization necessitates individual cloning, delivery, and coordinated replication of all segments to establish a successful infection. A few nanoviruses, faba bean necrotic yellows virus, faba bean necrotic stunt virus and Iranian sophora yellow stunt virus have been successfully cloned and reconstituted [25,26,27]. Like many geminiviruses and other ssDNA viruses, these nanovirus infectious clones have been successfully constructed using full-length monomers, partial tandem repeats, or dimeric forms of each genomic segment. These formats have enabled efficient Agrobacterium-mediated delivery, replication, and systemic infection across multiple hosts.

Milk vetch dwarf virus (MDV) is a well-studied member of the genus *Nanovirus*, transmitted by *Aphis craccivora* in a circulative, non-propagative manner [28,29] and has emerged as a significant pathogen in East Asia [1]. Initially reported in Japan on forage legumes, it has since been detected in a wide range of hosts, including lily, garlic, papaya, and solanaceous plants: tomato and pepper, which highlights its adaptability and raises concerns about potential cross-family transmission [2,30,31,32,33]. In Korea, MDV has been associated with symptoms like stunting, leaf curling, and yellowing in infected plants [1]. Its expanding host range underscores the importance of developing molecular tools such as ICs to better understand its replication and pathogenesis. To study the biology, host adaptation, and replication mechanisms of MDV, we constructed full-length ICs of MDV and tested their infectivity in *Nicotiana benthamiana*, *Nicotiana tabacum*, *Vicia faba*, and *Vigna unguiculata*. Additionally, we quantified the relative abundance of individual MDV segments in *N. benthamiana* and *V. faba* to evaluate their accumulation profiles during infection.

## 2. Materials and Methods

### 2.1. Virus Source and Nucleic Acid Extraction

The genetic material used in this study for constructing ICs of MDV was isolated from symptomatic papaya (*Carica papaya*) plants collected in Yesan, Korea, during 2017–2018. Total nucleic acids were extracted from infected leaf tissues using the Viral Gene-spin DNA/RNA Extraction Kit (iNtRON Biotechnology, Seongnam, Republic of Korea), following the manufacturer’s instructions. The extracted DNA served as the template for downstream amplification, cloning, and infectious clone assembly. The isolate, designated YS-AA-1, was partially characterized in our previous studies [2,30], and the complete genome has been submitted to GenBank under accession numbers MK726377, MK726376, MG852090, PV478020, PV478021, PV478022, PV478023, and PV478024.

### 2.2. Strategy for Infectious Clone (IC) Construction of MDV

To generate a tandem dimer-based infectious clone (IC) for nanovirus segments, a modular cloning strategy was employed, designed to yield a final construct with the genomic orientation IR-ORF-IR. Two overlapping fragments of the viral genome, designated IC1 and IC2, were PCR-amplified using primers engineered with specific restriction enzyme recognition sites at their termini. The primers designed for each MDV segment, incorporating flanking restriction sites, are listed in Table 1. Restriction sites at the 5′ end of IC1 and the 3′ end of IC2 were chosen to be unique and absent from the native viral sequence, ensuring specificity and enabling directional cloning. Conversely, the 3′ end of IC1 and the 5′ end of IC2 were engineered with the same restriction site, allowing seamless ligation of the overlapping region. This common site was strategically placed within the ORF region, enabling efficient reconstitution of a partial tandem repeat of the viral segment.

**Figure 1:**
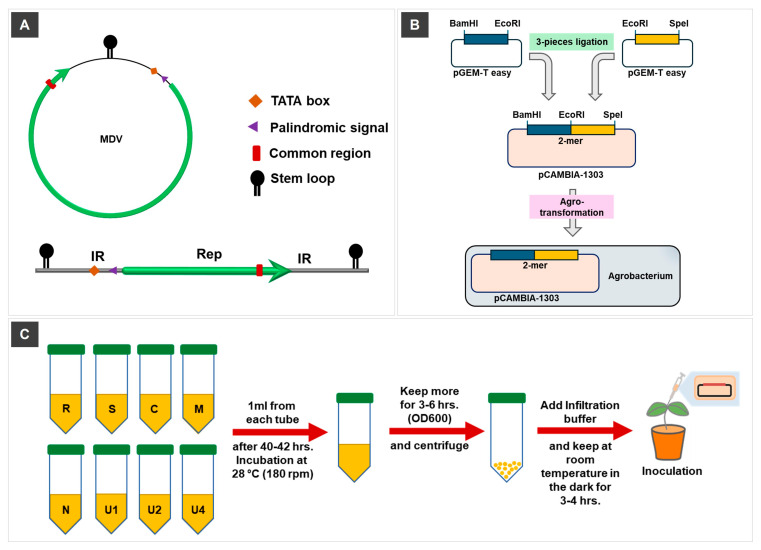
Schematic representation of the construction and delivery workflow of MDV-ICs. (**A**) Diagram of a single MDV genome segment with characteristic features including the intergenic region (IR), open reading frame (ORF), and segment-specific markers. (**B**) Cloning strategy for constructing tandem-repeat (2-mer) infectious clones. Two overlapping halves of each segment were amplified and assembled into a tandem dimer using a three-piece ligation method, combining two genome copies in head-to-tail orientation. The assembled 2-mer construct was inserted into the binary vector pCAMBIA1303 and introduced into *A. tumefaciens* via transformation. This design ensures efficient replication upon plant delivery and mimics the natural structure of replicating viral intermediates. (**C**) Workflow depicting the preparation of Agrobacterium cultures containing infectious clones of all eight MDV genomic segments (R, S, C, M, N, U1, U2, U4), culture mixing, centrifugation, and resuspension in infiltration buffer, followed by agroinoculation into host plants.

Each PCR product was initially cloned into the pGEM^®^-T Easy vector (Promega, Madison, WI, USA) to facilitate sequence verification and efficient subcloning. The verified IC1 and IC2 clones were then double-digested using the corresponding restriction enzymes. The overlapping digested fragments were gel-purified and assembled through three-piece ligation with a similarly digested pCAMBIA1303 binary vector, resulting in a plasmid containing the full-length viral segment flanked by duplicated intergenic regions. This construct was then introduced into *Agrobacterium tumefaciens* GV3101 using heat shock transformation for subsequent agroinoculation into host plants. A detailed schematic representation of the IC construction for MDV segments is shown in Figure 1.

### 2.3. Agrobacterium Transformation and Agroinoculation Procedure

The presence of the recombinant binary vector in *A. tumefaciens* was confirmed by colony PCR using segment-specific primers. Positive *A. tumefaciens* colonies were cultured in Luria–Bertani (LB) broth supplemented with kanamycin (50 mg/L), gentamicin (50 mg/L), and rifampicin (25 mg/L) at 28 °C with shaking (180 rpm) for 42–44 h. Equal volumes (1 mL) from each segment-specific culture were pooled into a fresh 50 mL Falcon tube and incubated at 28 °C for an additional 2–3 h until the optical density at 600 nm (OD_600_) reached approximately 0.6. After centrifugation at 5000× *g* for 10 min at room temperature, the bacterial pellet was resuspended in infiltration buffer containing 10 mM MES (pH 5.6), 10 mM MgCl_2_, and 100 µM acetosyringone, and kept in the dark for 3–4 h at room temperature to induce virulence gene expression. Inoculation was performed using the pinprick method [34] with three replicates of twelve plants each for *N. benthamiana* and *V. faba* (36 plants per species in total), and two replicates of unequal size (12 and 6 plants) for *V. unguiculata* and *N. tabacum* (18 plants per species in total). Three mock-inoculated plants were included as negative controls for each host in every replicate.

### 2.4. Plant Maintenance and Segment Reconstitution Analysis by PCR

Following agroinoculation, the host plants were maintained in a controlled growth chamber in Sungkyunkwan University, Suwon and Gyeongkuk National University, Andong, Korea, under a 16 h light/8 h dark photoperiod, with temperatures set at 28 °C during the light phase and 25 °C during the dark phase. Systemic leaves were harvested at 28 dpi from each host. For *V. unguiculata*, the third to fourth leaf above the point of inoculation was collected. In the case of *N. benthamiana*, *V. faba* and *N. tabacum* an upper fully expanded systemic leaf was sampled. Total DNA was extracted from systemic leaves of the inoculated plants using the Viral Gene-spin DNA/RNA Extraction Kit (iNtRON Biotechnology, Seongnam, Republic of Korea), following the manufacturer’s instructions and subjected to PCR amplification. Segment-specific PCR was performed using primer sets designed for each MDV genomic component (Table 2) to confirm the presence and replication of the viral segments.

PCR reactions were carried out in a final volume of 20 μL, containing 20 ng of template DNA, 1× AccuPower PCR Master Mix (Bioneer, Daejeon, Republic of Korea), and MDV segment-specific primers. Amplifications were performed using a T100™ Thermal Cycler (Bio-Rad, Hercules, CA, USA) under the following cycling conditions: initial denaturation at 94 °C for 3 min; followed by 35 cycles of denaturation at 94 °C for 30 s, annealing at 58 °C for 30 s, and extension at 72 °C for 1 min; with a final extension at 72 °C for 10 min. Amplified PCR products were visualized on 1% agarose gels stained with ethidium bromide and documented using a gel documentation system. Selected amplicons were purified and sent for sequencing (Macrogen, Seoul, Republic of Korea) to confirm segment identity.

### 2.5. Quantification of Segment Abundance by qPCR and Fold Change Analysis

Quantitative PCR (qPCR) was performed using total DNA extracted from systemic leaves of agroinoculated *N. benthamiana* and *V. faba* plants at 28 days post-inoculation. The objective was to quantify the relative accumulation of each viral genomic segment. Segment-specific primers were used for each of the eight MDV segments as shown in Table 3, and the elongation factor 1-alpha (*EF1α*) gene was used as an internal reference for normalization.

Each qPCR reaction was set up in a 20 µL volume containing 10 ng of total DNA (adjusted by dilution to ensure consistent concentration across all samples), 10 µL of 2× SYBR Green Master Mix (Takara Bio Inc., Shiga, Japan), 0.4 µL of each primer (10 µM), and nuclease-free water. Reactions were conducted in technical triplicate using a CFX96 Real-Time PCR Detection System (Bio-Rad, USA) with the following cycling conditions: initial denaturation at 95 °C for 3 min, followed by 40 cycles of denaturation at 95 °C for 10 s and annealing/extension at 60 °C for 30 s. Melt curve analysis was performed using CFX Maestro Software 2.2 (Bio-Rad). Cycle threshold (Ct) values for each MDV genome segment were normalized to the endogenous reference gene *EF1α* using the ΔCT method. Relative abundance levels of MDV segments in *N. benthamiana* and *V. faba* were calculated using the 2^−ΔΔCT^ method [35]. EF1α was used as the internal reference gene, and mock-inoculated plants served as calibrators for each host. For host-wise comparisons, the resulting 2^−ΔΔCT^ values were log_10_-transformed to normalize the scale. Three plants per host were analyzed, each with three technical replicates. Segment-wise abundance profiles were visualized using dotted line plots with distinct markers to enable clear comparison between hosts.

## 3. Results

### 3.1. Reconstitution of MDV Segments Through PCR

**Figure 2:**
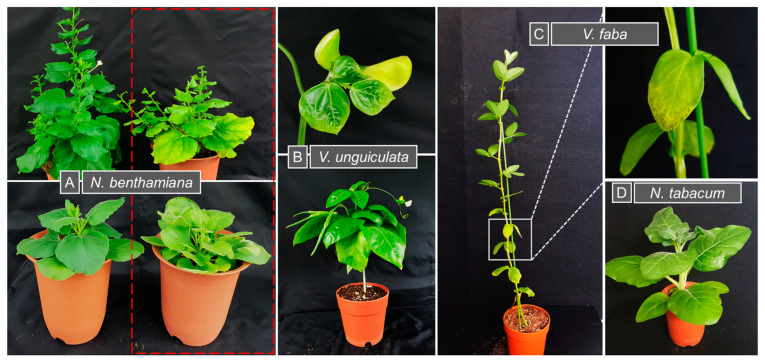
Symptom development in host plants infected with MDV at 28 dpi. Plants were agroinoculated with infectious clones comprising all MDV genomic segments. (**A**) MDV infected *N. benthamiana* plants (**right**, red-dashed box) showing stunting, leaf yellowing and bushy growth compared with negative controls (**left**). (**B**) *V. unguiculata* showed stunting along with crinkling, mild leaf curling. The top panel shows symptomatic young leaves, while the bottom panel depicts whole plant symptoms. (**C**) *V. faba* infected plants developed necrotic lesions accompanied by leaf yellowing, as shown in symptomatic leaves and (**D**) *N. tabacum* displayed only mild stunting.

**Figure 3:**
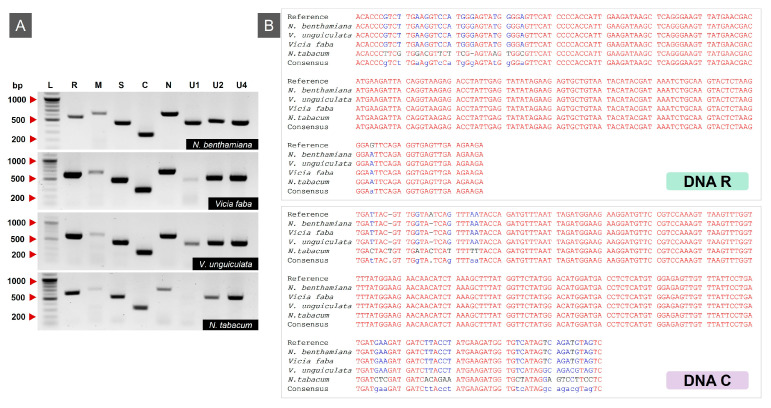
Detection and sequence variation in MDV segments in different host plants. (**A**) PCR amplification of MDV segments (R, M, S, C, N, U1, U2, and U4) from four infected hosts: *N. benthamiana*, *V. faba*, *V. unguiculata*, and *N. tabacum*. Segment-specific bands were detected in all tested hosts, confirming systemic infection and segment presence. Lane L: Bioneer 100 bp ladder; segment sizes range from ~200 to ~700 bp. (**B**) Nucleotide sequence alignments of cloned MDV DNA-R and DNA-C segments from each host compared with the reference sequence. Minor nucleotide substitutions (highlighted in blue/purple) were observed, particularly in samples from *N. tabacum*. Consensus sequences are shown at the bottom of each alignment panel.

Following agroinoculation with ICs of all eight MDV genomic segments, all host species: *N. benthamiana*, *V. unguiculata*, *V. faba* and *N. tabacum* were maintained under controlled growth conditions. By 28 days post-inoculation (dpi), visible disease symptoms were observed; *N. benthamiana* displayed both stunting and leaf yellowing, whereas *V. unguiculata* showed stunting along with crinkling, mild leaf curling and yellowing. *V. faba* developed necrosis and yellowing while *N. tabacum* displayed only mild stunting without distinct additional symptoms (Figure 2). These phenotypic responses indicated successful systemic infection in host plants. Viral DNA was amplified from all agro-inoculated samples using segment-specific PCR, yielding amplicons of the expected sizes (Figure 3A). No bands or amplified products were detected in the negative controls. PCR products corresponding to all eight genomic segments were then purified and sequenced from each host to confirm segment identity. Among these, only the sequencing results of segments R and C are presented here (Figure 3B). Among all MDV segments, only DNA-M and DNA-U1 were not present in all samples tested and mainly in *V. unguiculata* and *N. tabacum* plants. A summary of the number and percentage of samples exhibiting successful segment detection is provided in Table 4.

### 3.2. Relative Accumulation of MDV Segments in Infected Plants

**Figure 4:**
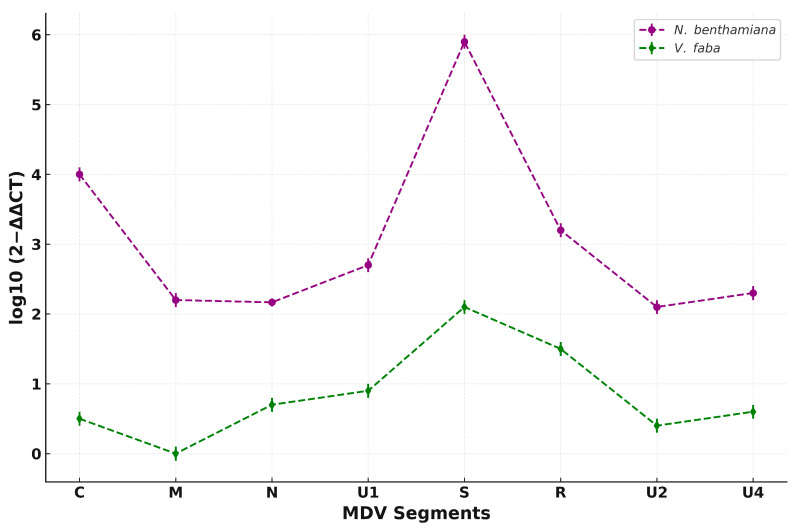
Relative abundance of MDV genomic segments in *N. benthamiana* and *V. faba*. Segmental DNA levels were quantified using the 2^−ΔΔCT^ method. Data represent the mean of three biological replicates per host; each performed in three technical replicates. Dotted lines with distinct markers illustrate differences in segment-wise accumulation between the two hosts. Overall, higher viral DNA accumulation was observed in *N. benthamiana*, with segment S showing the highest abundance among all segments in both hosts.

To compare the relative abundance of MDV segments in two different hosts, 2^−ΔΔCT^ values were calculated from qPCR data obtained from *N. benthamiana* and *V. faba* and subsequently log_10_-transformed for host-wise comparison. Overall, all MDV segments accumulated to higher levels in *N. benthamiana* than in *V. faba*. Among them, DNA-S exhibited the highest abundance in both hosts, with a pronounced peak in *N. benthamiana*. DNA-R, DNA-C, and DNA-U1 also showed strong accumulation in *N. benthamiana*, whereas DNA-N and DNA-U4 were detected at moderate levels. In contrast, DNA-M and DNA-U2 represented the least accumulated segments in both hosts, with DNA-M showing particularly low levels in *V. faba*. Interestingly, although absolute abundance levels varied significantly between hosts, the relative ranking of individual segments remained largely consistent, as reflected by the parallel trends in the segment-wise abundance profiles. This suggests that segment-specific accumulation patterns are conserved, while the host influences the overall magnitude of replication. The comparative data are presented in Figure 4.

## 4. Discussion

The development of ICs has been pivotal in plant virology, offering a reliable and reproducible means to reconstitute viral infections under controlled conditions [17,24,36,37]. Particularly for insect-transmitted viruses, which cannot be mechanically inoculated, ICs not only facilitate experimental infection without relying on natural vectors but also reduce the risks of genetic drift and mixed infections associated with repeated virus passage in host plants [20,21,22]. In this study, we constructed full-length tandem-dimer-based ICs of all eight genomic segments of MDV to investigate its replication, segmental accumulation, and suitability for cellular-level assays. Unlike monopartite viruses such as geminiviruses, successful reconstruction of a nanovirus infectious system requires simultaneous delivery and replication of all essential segments within the host cells, though not in the same cell [5]. All MDV segments were successfully cloned into a binary vector under a tandem repeat configuration to promote efficient replication after agroinoculation. The use of tandem dimers or constructs of greater-than-unit-length has been shown to enhance infectivity and systemic movement in ssDNA viruses [38,39]. Segment-specific primers incorporating restriction sites facilitated modular assembly of the constructs and improved downstream validation. Our strategy ensured that each segment could be independently cloned and later mixed for co-delivery.

To evaluate the functionality of the constructed clones, the complete segment set was introduced into host plants using *Agrobacterium*-mediated inoculation. *N. benthamiana* was selected as the primary host for clone delivery due to its high transformation efficiency, well-established use in transient expression assays, and susceptibility to a broad range of plant viruses [40,41]. Although *N. benthamiana* is not a natural host of MDV, it supports viral replication and serves as a tractable platform for initial validation. Following agroinoculation, PCR confirmed the successful reconstitution of all eight MDV segments from systemically infected leaves. The same strategy was extended to three additional hosts of nanoviruses, *V. unguiculata*, *V. faba*, and *N. tabacum* [42], where systemic infection was also confirmed, supporting the utility of the constructed clones for cross-host validation.

We successfully reconstituted MDV in all four host species and confirmed that the DNA-R and DNA-C sequences from *N. benthamiana*, *V. unguiculata*, and *V. faba* were identical to the reference. In contrast, *N. tabacum* showed distinct sequence variations in DNA-R and DNA-C (Figure 3B). While MDV has been reported previously in *N. tabacum*, the sequence divergence we observed may reflect host-specific constraints on replication fidelity or adaptive changes that emerge during infection. Although the name of MDV implies a primary association with dwarfing, our results revealed a broader spectrum of symptoms across host plants. In addition to stunting, we commonly observed leaf and vein yellowing, upward curling, and mild crinkling. Interestingly, necrosis appeared only in *V. faba*, which may reflect host-specific symptom expression (potentially influenced by this species’ known sensitivity to other necrosis) inducing viruses like FBNYV. One of the most striking and consistent patterns was the development of a bushy phenotype, which appeared in nearly all infected *N. benthamiana* and *V. unguiculata* plants, highlighting a possibly conserved response to MDV infection across diverse hosts.

To compare MDV segmental accumulation across host species, 2^−ΔΔCT^ values were calculated and log_10_-transformed to enable direct comparison between *N. benthamiana* and *V. faba*. While total accumulation levels were markedly higher in *N. benthamiana*, the relative ranking of segment abundance remained largely consistent between the two hosts. This was evident from the parallel segment-wise profiles, suggesting that MDV maintains a conserved segmental stoichiometry regardless of host, but the overall magnitude of accumulation is host-dependent.

These results suggest that MDV genome components are not maintained at equimolar levels during infection. While previous studies have reported non-equimolar accumulation patterns in nanoviruses, they were generally limited to a single host species. In contrast, our analysis across *N. benthamiana* and *V. faba* reveals that both the magnitude and the relative hierarchy of segment accumulation can vary between hosts, indicating that host-specific factors influence segment stoichiometry during infection [43,44].

In conclusion, this study presents a comprehensive framework for the construction and functional validation of full-length ICs for MDV. Segment-specific detection and quantification not only confirm the functionality of the clones but also provide important insights into the non-equimolar accumulation patterns of MDV genome components. These findings offer a valuable foundation for future studies aimed at unraveling the molecular determinants of segment compatibility, replication dynamics, and genome regulation in multipartite plant DNA viruses.

## Figures and Tables

**Table 1:** Primer sequences and corresponding restriction sites used for amplification and cloning of MDV genomic segments for infectious clone (IC) construction.

Segment	Primer Name	Restriction Site	Sequences (5′ *→* 3′)	Primer Name	Restriction Site	Sequences (5′ *→* 3′)	Product Size (bp)
MDV-C	IC1-F	*Bam*HI	**GGATCC**TATATTAAGTTG TTATCTGAGAAATCTATT	IC1-R	*Eco*RI	**GAATTC**AACTCAGCAGGTGAAG	634
	IC2-F	*Eco*RI	**GAATTC**GTTAAGTA AGTTTTTAAATGCTs	IC2-R	*Spe*I	**ACTAGT**TTTCGTTGTA AGAACAACGAAGAAA	810
MDV-R	IC1-F	*Kpn*I	**GGTACC**CGTCATAT GATCCCGTGCT	IC1-R	*Eco*RI	**GAATTC**GAACTCCCT TAGAGTACTTGC	557
	IC2-F	*Eco*RI	**GAATTC**AGAGGTG AGTTGAAGAAGA	IC2-R	*Spe*I	**ACTAGT**ATTTTATTGATGAATG ATAAAATATTACAACTTG	584
MDV-M	IC1-F	*Kpn*I	**GGTACC**AGAATGATTATA GATTGTAATTAGTTATTC	IC1-R	*Eco*RV	**GATATC**GCCGTCGTCTTGATA	698
	IC2-F	*Eco*RV	**GATATC**GATGCCCAGAAGAG	IC2-R	*Spe*I	**ACTAGT**GAAATTTCAAT GGACAATAAAAACAC	953
MDV-S	IC1-F	*Kpn*I	**GGTACC**TGTAATGAAGAACAC TATGAAATAATGAAACC	IC1-R	*Pst*I	**CTGCAG**ACCAATTAACAA TGGGAGAA	767
	IC2-F	*Pst*I	**CTGCAG**CTTTTACCGCTCC	IC2-R	*Bam*HI	**GGATCC**TTTCGTTGTG AGTACAACGAATAC	720
MDV-U1	IC1-F	*Bam*HI	**GGATCC**TAATGAATATTTGTTT CAGGATCAAACA	IC1-R	*Psi*I	**TTATAA**AAAACATTCTAATA CCTATCAAATAA	888
	IC2-F	*Psi*I	**TTATAA**ATATTAATCAGTTG ATTAATACTTGT	IC2-R	*Spe*I	**ACTAGT**GACCTCAATA GAAGCTTTAGTTTG	655
MDV-N	IC1-F	*Bam*HI	**GGATCC**TAATCATAATTATTGT AAGATTATGCAATTG	IC1-R	*Sca*I	**AGTACT**GGGATTC AATATCAAGGT	839
	IC2-F	*Sca*I	**AGTACT**TGAAGAAG GACGAAGAC	IC2-R	*Spe*I	**ACTAGT**TTTTTGCAGTTGCA GAAAATGATGAC	621
MDV-U2	IC1-F	*Bam*HI	**GGATCC**TGTGATATATGAAAAC AATTTGTTGTTTTTTCCATTG	IC1-R	*Eco*RV	**GATATC**TATAATTACCTGAA TCGTACAAATCTTTCAAG	930
	IC2-F	*Eco*RV	**GATATC**AAGTGTATTATTC TTCGTCATGTAAAAGAG	IC2-R	*Spe*I	**ACTAGT**AGAACAAGA ACGAGACTAACGC	464
MDV-U4	IC1-F	*Bam*HI	GAGCAATAACAAGAATAAATA A**GGATCC**AAATGCAA	IC1-R	*Sal*I	**GTCGAC**ATCTTCA AAGGGATTCTT	876
	IC2-F	*Sal*I	**GTCGAC**CCTGATGTTACC	IC2-R	*Spe*I	GTGGGGACCAT**ACTAGT** TTCTCACTTATTA	551

**Table 2:** Specific PCR primers for MDV segment detection purposes.

Experiment	Segment	Primer Name	Sequences (5′ *→* 3′)	Product Size (bp)
Reconstitution after agro-inoculation	MDV-C	C-Forward	CCTGCTGAATTGAATTCTCTGAGTA	295
		C-Reverse	AAACTATCTGAATACCTAGCGACTTAAAC	
	MDV-S	S-Forward	CTGCTTTGTTGAAGAAAGATGAAGTC	488
		S-Reverse	AAACACGGAAACATACCGCTAC	
	MDV-N	N-Forward	GAAGCTTCTTCGTTGCTCTATAAATACAAG	673
		N-Reverse	TCAGATGACGTCATATTCATTTGGG	
	MDV-M	M-Forward	CCTGAGCCGCTATTGTCAT	672
		M-Reverse	TTCCTCATTGGCTACTGAATTGG	
	MDV-R	R-Forward	GAGATGAAGAAACGCACGTCT	526
		R-Reverse	GCACTAACTCTTGGTGGTC	
	MDV-U1	U1-Forward	CGTCTGAGAGGAAATTGATAGCC	486
		U1-Reverse	GGGCCTAGACATATAGCTTCG	
	MDV-U2	U2-Forward	CGAGCGTTAGTCTCGTTCTTG	516
		U2-Reverse	TGTTATCAATTGTAGTTGTCTTCCACC	
	MDV-U4	U4-Forward	CCACGCACTATATGAACCTTGC	516
		U4-Reverse	GCAAATATTGAAGGTCTTCACCATC	

**Table 3:** qPCR primers used for MDV detection in this Study.

Segment	Primer Name	Sequences (5′ *→* 3′)	Product Size (bp)
MDV-M	MDVQ-M-F	GCCCAGAAGAGACATCAAGC	196
	MDVQ-M-R	CGAAGGGTGTGCGTGTTATAG	
MDV-U4	MDVQ-U4-F	ATGGA ACCCAGGTTCCTTCTT	163
	MDVQ-U4-R	TCCTCTGGTTGTTCAAACGTAT	
MDV-N	MDVQ-N-F	GAAGGTCAGAAGACATTCAACCT	159
	MDVQ-N-R	ACACTTTGATCCTAAGAGCATG	
MDV-R	MDVQ-R-F	GGCTTAGTATTACCCCCGCC	137
	MDVQ-R-R	GCACCAGCATATAACTTGCCG	
MDV-C	MDVQ-C-F	AATACGCGTGGACGATCAGG	177
	MDVQ-C-R	CGGGAAGAAGCAAAGACAGC	
MDV-S	MDVQ-S-F	CCGGTATCAGCCAAACCCAA	164
	MDVQ-S-R	ATACCGCTACGCGGAGTTTT	
MDV-U1	MDVQ-U1-F	CTTCGTCTCGAAGCAAAGGAC	145
	MDVQ-U1-R	TCGTTCGCAGACATAACCTCAA	
MDV-U2	MDVQ-U2-F	AAGGAAGAACAAGATGCTTTCTGG	150
	MDVQ-U2-R	TCTAAGAACCCACCGTGCAG	

**Table 4:** Summary of symptoms and segment detection in MDV-inoculated host plants at 28 dpi.

Host Species	Symptoms Observed *	DNA-R	DNA-S	DNA-C	DNA-M	DNA-N	DNA-U1	DNA-U2	DNA-U4
*N. benthamiana*	Bushy growth Stunting and leaf yellowing	36/36	33/36	35/36	31/36	36/36	32/36	33/36	34/36
*V. unguiculata*	Leaf yellowing	18/18	18/18	15/18	14/18	18/18	14/18	16/18	12/18
*V. faba*	Necrosis, leaf yellowing, curling	36/36	33/36	35/36	23/36	36/36	30/36	33/36	34/36
*N. tabacum*	Mild stunting	18/18	18/18	15/18	07/18	18/18	03/18	16/18	12/18

* In most cases, plants lacking detectable levels of DNA-U1 did not exhibit either symptoms or showed only mild symptoms.

## Data Availability

Data is contained within the article: The original contributions presented in this study are included in the article. Further inquiries can be directed to the corresponding authors.
